# pH-Responsive Bacterial Nanocellulose Smart Labels Derived from Acid Whey for Estimating Beef Mince Quality Alterations During Storage

**DOI:** 10.3390/foods14091544

**Published:** 2025-04-28

**Authors:** Dylan Zhe Liu, Sabeen Hassan, Benjamin M. Long, Alan Labas, Jayendra K. Amamcharla, Michelle J. Y. Yoo, Xiaojie Hu, David C. Bean

**Affiliations:** 1 Department of Food Science and Human Nutrition, Iowa State University, Ames, IA 50011, USA; 2 Institute of Innovation, Science, and Sustainability, Federation University Australia, Mount Helen, VIC 3350, Australia; sabeenhassan101@gmail.com (S.H.); bm.long@federation.edu.au (B.M.L.); a.labas@federation.edu.au (A.L.); xiaojieh@students.federation.edu.au (X.H.); d.bean@federation.edu.au (D.C.B.); 3 Midwest Dairy Foods Research Center, Department of Food Science and Nutrition, University of Minnesota, St. Paul, MN 55108, USA; jayendra@umn.edu; 4 School of Science, Faculty of Health and Environmental Sciences, Auckland University of Technology, Auckland 1010, New Zealand; michelle.yoo@aut.ac.nz

**Keywords:** nanocellulose, real-time food quality indicators, colorimetric smart label, anthocyanin, acid whey

## Abstract

This study develops a pH-responsive label by incorporating anthocyanin from *Clitoria ternatea* into a bacterial nanocellulose (BNC) film derived from acid whey fermentation. The labels were designed to display two distinct colors—pink and purple—by adjusting the pH of anthocyanin and were integrated into beef mince packaging to monitor quality changes over a 15-day storage period at 4 °C. Color variations were assessed using a chroma meter and visual inspection, with both label types exhibiting a shift to blue in response to a deterioration in freshness. Significant differences (*p* < 0.05) in total color difference (∆E) were observed across data collection days. The pink label showed an ∆E of 14.19 between day 0 and day 8, increasing to 27.39 by day 15. The purple label exhibited an ∆E of 12.94 by day 8 and 27.86 by day 15. A Total Volatile Basic Nitrogen (TVBN) analysis and microbial evaluations confirmed a degradation in the quality of the beef mince, with strong correlations between ∆E and ∆TVBN (r = 0.956 for pink, r = 0.993 for purple). Additionally, good correlations were recorded between label total color differences and coliform counts (r = 0.933 for pink, r = 0.875 for purple), as well as Total Plate Counts (TPCs) (r = 0.982 for pink, r = 0.950 for purple). These results highlight the potential of acid whey-derived nanocellulose films as real-time quality indicators for intelligent food packaging systems.

## 1. Introduction

The global food industry faces significant challenges in ensuring food quality, safety, and sustainability. One of the primary concerns is the rapid spoilage of perishable foods, which contributes to food waste and economic losses. Intelligent packaging technologies, particularly real-time food quality indicators, have emerged as a promising solution to address these challenges. These indicators allow the continuous monitoring of food’s freshness and environmental conditions, enabling consumers and supply chain stakeholders to make informed decisions without breaching the integrity of the packaging. Freshness labels, a subset of intelligent packaging, can detect spoilage markers such as pH changes, gas emissions, and microbial activity, thereby improving food’s traceability and minimizing unnecessary food waste [1,2,3,4].

Among the various spoilage indicators, pH variations are particularly significant in meat, seafood, and dairy products, as they are highly susceptible to microbial activity and biochemical degradation. As food deteriorates, metabolic by-products alter its pH, making pH-sensitive intelligent labels a reliable tool for real-time freshness assessments. These labels typically comprise two essential components: a support material and a pH-sensitive indicator.

Nanocellulose has gained considerable attention as a sustainable material for intelligent packaging due to its exceptional properties, including biodegradability, non-toxicity, and high mechanical strength [5,6,7,8]. Among different nanocellulose sources, bacterial nanocellulose (BNC) has been recognized for its homogeneous structure, high purity, and ability to serve as an ideal carrier for functional additives. Notably, BNC derived from the symbiotic culture of bacteria and yeast (SCOBY), traditionally used in kombucha fermentation, has demonstrated a high potential for intelligent packaging applications [9,10,11].

However, the conventional fermentation process for SCOBY-based BNC production is time-intensive, often requiring 7 to 21 days depending on the culture conditions and substrate composition [12,13]. To enhance production efficiency and reduce costs, this study explores the utilization of acid whey—a dairy by-product—as an alternative fermentation medium. Acid whey, a residual by-product from cheese and yogurt production, is rich in nutrients that can support microbial growth, while simultaneously promoting a circular economy approach by upcycling food-processing waste [14]. Significantly, this study utilizes food waste (acid whey) to produce intelligent packaging applications that can further reduce food waste through the value chain.

Incorporating pH-sensitive natural pigments into intelligent labels provides a safe and visually interpretable approach to monitoring food’s freshness. Synthetic pH indicators, while effective, are increasingly scrutinized due to potential toxicity concerns and regulatory restrictions [15,16,17,18,19]. As a natural alternative, anthocyanins—flavonoid pigments found in various fruits, vegetables, and flowers—offer an environmentally friendly and food-safe solution. Anthocyanins exhibit distinct color changes in response to pH fluctuations, making them suitable candidates for real-time freshness monitoring [20,21,22,23,24]. In this study, anthocyanins from *Clitoria ternatea* (butterfly pea flower) were selected as the pH-responsive component. These pigments transition from pink-red to dark green over a pH range of 2 to 11, providing a clear and effective indication of food spoilage [22,23,25]. When integrated with the whey-derived nanocellulose substrate, this system creates a sustainable, functional freshness indicator that is robust, non-toxic, and biodegradable.

This study pioneers a novel approach to intelligent food packaging by integrating anthocyanin-based pH indicators with nanocellulose films derived from acid whey fermentation. By upcycling dairy by-products into functional packaging materials, this research not only enhances food safety and consumer awareness but also aligns with the principles of the circular economy. The proposed system has the potential to mitigate food waste; reduce reliance on synthetic additives; and promote sustainable, environmentally friendly, safe, and cost-effective material development in the food packaging sector.

## 2. Materials and Methods

### 2.1. Materials

Acid whey, comprising a mixture of whey derived from the production of various cheese types, was collected from a cheese-manufacturing facility in Melbourne, Australia. Upon collection, it was immediately frozen and stored at −18 °C before use. The symbiotic culture of bacteria and yeast (SCOBY) was sourced from The Good Brew Company (Melbourne, Australia). Reagent-grade sucrose was procured from Merck and used as the primary carbon source for microbial fermentation. Yeast Extract was obtained from Thermo Fisher Scientific (Waltham, MA, USA).

### 2.2. Preparation of BNC Material via Static Fermentation

BNC films were synthesized following a previously established protocol [14] with slight modifications. Acid whey was thawed at an ambient temperature (22 ± 2 °C) and heated to 95 °C for 5 min with the addition of 2.5% (*w*/*w*) sucrose, ensuring complete dissolution. Separately, a 10% (*w*/*w*) Yeast Extract solution was prepared by heating it at 95 °C for 5 min and subsequently mixed with the acid whey solution to achieve a final Yeast Extract concentration of 0.3% (*w*/*w*). After cooling to room temperature (22 ± 2 °C), the medium was inoculated with a 20% (*w*/*w*) SCOBY culture and incubated under static aerobic conditions at 22 ± 2 °C. Following a 5-day fermentation period, the BNC films were carefully collected from the surface of the medium.

### 2.3. Anthocyanin Extraction from Clitoria ternatea

The extraction procedure was adapted from previously established protocols [25,26,27,28] with specific modifications. Twenty grams of dried *C. ternatea* flowers (Wild Hibiscus Flower Company, Richford, VT, USA) was blended with 250 mL of reverse osmosis (RO) water at room temperature to initiate anthocyanin extraction. The mixture was then transferred to a 50 °C water bath for 30 min, stirred intermittently, and covered with aluminum foil to minimize evaporation. After incubation, the extract was filtered using Whatman Grade No. 1 filter paper to remove plant residues, and the resulting filtrate was stored at room temperature for subsequent applications. For further processing, the extracted dye was diluted 1:4 with RO water, followed by pH adjustment to induce color variations. The pH was modified using 1M hydrochloric acid, which caused the anthocyanin solution to transition from blue to pink at pH 2.0 and purple at pH 3.0. The pH variation was carefully monitored with a pH meter (LAQUAtwin pH-11, HORIBA Advanced Techno, Co., Ltd., Kyoto, Japan) to ensure accurate colorimetric transitions for pH-responsive labeling applications. The extracted anthocyanin was freeze-dried using a freeze dryer (Alpha 2-4 LD Plus, Christ, Hagen, Germany) at −32 °C to facilitate Fourier-Transform Infrared (FTIR) spectroscopy analysis.

### 2.4. Determination of Total Anthocyanin Content (TAC)

The quantification of anthocyanin in the *C. ternatea* extract was conducted using the pH differential method described previously [22,29], with modifications. A 0.5 mL aliquot of the extract was mixed with 4.5 mL 0.4 M sodium acetate (pH 4.5) and 0.025 M potassium chloride (pH 1.0), following the protocol described by a previous study [30]. The absorbance of the prepared solutions was measured at 520 nm and 700 nm using a UV–VIS spectrophotometer (UVmini-1240, Shimadzu Corporation, Kyoto, Japan), with RO water serving as the reference baseline. The TAC was then calculated and expressed as mg cyanidin-3-glucoside per mL using the established equations:

(1) $$A={\left(A_{520}-A_{700}\right)}_{\mathrm{pH}=1}-{\left(A_{520}-A_{700}\right)}_{\mathrm{pH}=4.5}$$

(2) $$\mathrm{TAC}=\frac{A\times M_{W}\times 100}{\mathrm{MA}}$$

where A represents the absorbance, MA denotes the molar absorptivity of cyanidin-3-glucoside at 26,900, and M_W_ is the molecular weight of cyanidin-3-glucoside, which is 449.2 g/mol.

### 2.5. Preparation of pH-Responsive Labels

Anthocyanin was extracted from *C. ternatea*, resulting in a total anthocyanin content of 0.589 mg/L. The anthocyanin extract was diluted with RO water at a ratio of 1:4 before adjusting the pH for subsequent applications. The diluted anthocyanin dye solution was carefully adjusted to achieve an optimal concentration that could effectively indicate food quality changes through visible color transitions, ensuring the coloration was neither too dark nor too faint.

The wet BNC films were carefully retrieved from the surface of the fermentation medium and immediately weighed to ensure consistency in subsequent processing. The films were then mixed with RO water at a 1:20 mass ratio and subjected to heat treatment at 95 °C for 5 min to improve their dispersibility and remove residual impurities. Following heating, the films were allowed to cool to ambient temperature and then blended in a high-speed blender for 2 min to achieve a uniform dispersion.

For bleaching, the blended BNC suspension was combined with 30% (*w*/*v*) hydrogen peroxide (analytical reagent) at a 1:20 ratio and left to react in a sealed container for 48 h at room temperature. After the bleaching process, the BNC mixture was centrifuged at 4000× *g* at room temperature using a centrifuge (Universal 30F centrifuge, Hettich, Westphalia, Germany). The pellet was then resuspended in RO water, and the centrifugation process was repeated three times to ensure complete removal of the bleaching agent. The BNC was then evenly spread onto a silicone mat and oven-dried at 40 °C overnight to obtain thin, white cellulose films. Following drying, the cellulose films were submerged into the prepared pH-responsive anthocyanin dye solutions for 30 min at ambient temperature (22 ± 2 °C), resulting in a pink and purple coloration corresponding to the respective pH conditions. Subsequently, the dyed films were oven-dried again at 40 °C overnight. The final dyed BNC films exhibited colors consistent with the diluted anthocyanin solutions adjusted to corresponding pH levels, indicating no noticeable acid-base reactions during label preparation.

### 2.6. Fourier-Transform Infrared Spectroscopy (FTIR) Assessment

The FTIR spectroscopy analysis was conducted following the procedure described by [2], with slight modifications. Both unmodified BNC films and those integrated with anthocyanin were analyzed using a Frontier FT-IR Single-Range spectrometer (PerkinElmer Inc., Waltham, MA, USA). The infrared spectra were recorded over a wavenumber range of 4000 to 600 cm^−1^, with a spectral resolution of 4 cm^−1^. Each specimen underwent 16 consecutive scans to ensure reproducibility.

### 2.7. Indicator Implementation and Food Storage Evaluation

**Figure 1:**
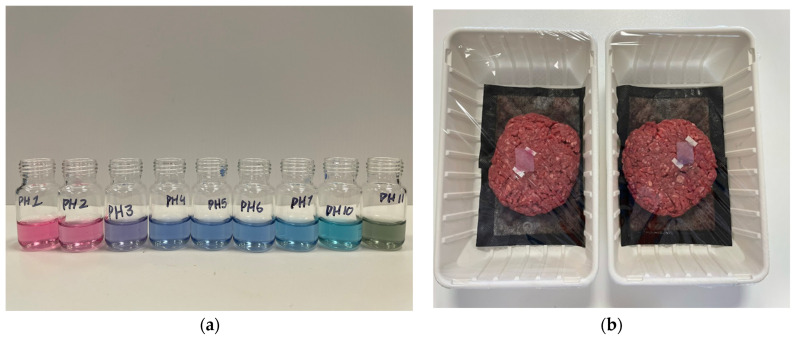
Monitoring beef mince Using a pH-responsive BNC label. (**a**) Color responses of anthocyanin solution (from left to right, the pH of the anthocyanin solutions was adjusted to pH 1.0, pH 2.0, pH 3.0, pH 4.0, pH 5.0, pH 6.0, pH 7.0, pH 10.0, and pH 11.0, respectively); (**b**) real-time freshness monitoring of beef mince using a pH-responsive BNC label.

Eight containers, each containing 250 g of extra-lean beef mince (approximately 5% fat content, purchased from a local supermarket), were accurately weighed before testing. The beef mince samples had 8 days remaining before the best-before date indicated on the label at the start of the experiment. The best-before date serves as a guideline for optimum quality, when stored correctly. Desiccated BNC labels were trimmed into 1.5 cm × 2 cm rectangular strips and affixed to the inner surface of the beef mince packaging, ensuring that the pH-responsive indicator did not come into direct contact with the food product (shown in Figure 1b). The label was placed centrally on top of the beef mince to ensure optimal contact with volatiles released from the beef sample and to maintain a consistent label positioning. A moisture-absorbing pad was placed beneath the beef mince to minimize the excess moisture accumulation throughout the 15-day storage period at 4 °C.

The packaged beef mince, along with the embedded indicators, was refrigerated at 4 °C for 15 days, with four replicates for each label type to ensure reproducibility. Observations and analyses of the beef mince samples were conducted on days 0, 3, 8, 10, and 15, assessing both microbiological attributes and the colorimetric responses of the pH-sensitive indicators. The color changes of the indicators were quantified using the methodology detailed in the subsequent section.

### 2.8. Color Response Assessment

A chroma meter (Konica Minolta Inc., Tokyo, Japan) was used to analyze the color responses of the pH-responsive labels, following a method adapted from [9], with slight modifications. Measurements were conducted under standardized lighting conditions and ambient temperature (22 ± 2 °C). The device was calibrated using a standard white calibration tile prior to each session. Four distinct points were selected for color evaluation to ensure a consistent measurement. The baseline color of each pH-responsive label was recorded before testing, and subsequent color changes were documented at designated storage intervals.

The colorimetric analysis was conducted using Hunter’s color indices, specifically L (lightness), a (red-to-green spectrum), and b (blue-to-yellow spectrum). The total color difference (∆E) was calculated using the following equation:

(3) $$\Delta E=\sqrt{[{\left(L{}^{\circ}-L\right)}^{2}+{\left(a{}^{\circ}-a\right)}^{2}+{\left(b{}^{\circ}-b\right)}^{2}}$$

where L°, a°, and b° represent the initial baseline color values, and L, a, and b denote the color values recorded after storage.

### 2.9. Measurement of Mince pH

A 5 g sample of beef mince was combined with 45 mL of RO water in a sterile stomacher bag. The mixture was homogenized using a stomacher for 60 s at a speed of 8 strokes per minute to ensure thorough blending. Following homogenization, the pH of the resulting suspension was measured using a calibrated pH meter (SevenDirect SD20 pH meter, Mettler Toledo, Greifensee, Switzerland), adjusted from a previous study [31].

### 2.10. Determination of Total Volatile Basic Nitrogen (TVBN) Content

The TVBN quantification of beef mince was conducted following the methodologies described previously [32,33], with slight modifications. A 10 g sample of beef mince was homogenized with 50 mL of RO water and transferred into a 500 mL round-bottom flask, followed by an additional 150 mL of RO water. The process then introduced 2 g of magnesium oxide and four drops of silicone antifoam agent. Steam distillation was performed using a Kjeldahl distillation system (K-355 Distillation system, Büchi Labortechnik AG, Flawil, Switzerland), with 125 mL of distillate collected in an Erlenmeyer flask containing 37 mL of 2% boric acid and 40 µL of a mixed indicator solution (methyl red and methyl blue). The solution was then titrated with 0.1 M hydrochloric acid until a color change from green to purple was observed.

The TVBN (mg/100 g) was calculated using the following equation:

(4) $$\mathrm{TVBN}=[V\times C\times (\frac{14}{m})]\times 100$$

(5) $$\Delta \mathrm{TVBN}={\mathrm{TVBN}}_{i}-{\mathrm{TVBN}}_{0}$$

where V represents the volume of hydrochloric acid added during titration (mL), C is the hydrochloric acid concentration (N), and m is the weight of the initial food specimen (g). ∆TVBN refers to the change in TVBN from day 0 to day *i*, where *i* represents the measurement day starting from day 3.

### 2.11. Microbiological Assessment

A 5 g sample of beef mince was combined with 45 mL of buffered peptone water in a sterile stomacher bag and homogenized using a stomacher for 60 s to ensure a uniform suspension. Serial dilutions of the homogenate were then prepared, ranging from 10^−1^ to 10^−^⁶, using sterile techniques. For each dilution, 100 µL of the sample was evenly spread onto Chromocult Coliform (Merck KGaA, Darmstadt, Germany) chromogenic agar plates and Total Plate Count (Oxoid Ltd., Hampshire, UK) agar plates using a sterile pipette. The plates were incubated at 37 °C for 24 h to facilitate microbial growth. To ensure accuracy and reproducibility, each dilution was plated in triplicate.

### 2.12. Statistical Evaluation

The experimental data were statistically analyzed using Microsoft Excel (Version 2208) and IBM SPSS Statistics 29. Data were evaluated using one-way ANOVA to determine significant differences between groups, followed by the Duncan test where necessary. Additionally, a Pearson correlation analysis was performed to assess the strength and direction of relationships between variables.

## 3. Results

### 3.1. Bacterial Nanocellulose Synthesis

BNC synthesis was conducted under static conditions, requiring sufficient aeration and an optimal temperature for bacterial growth. The efficiency of the BNC production depended on the carbon and nitrogen sources, as well as the composition of microorganisms present in the culture medium [2]. Following a five-day incubation period, the BNC film derived from acid whey exhibited an average wet weight yield of 31.27 ± 9.60 g/L, a dry weight yield of 1.14 ± 0.39 g/L, and a wet film thickness of 2.75 ± 0.35 mm (Table 1). An extended cultivation time has been shown to increase the BNC film thickness [34]. The morphological characteristics of the BNC film, as reported in our previous study [14], revealed a high surface area with abundant pores and tunnels. These structural features enhance the capacity of the BNC film to incorporate pH-responsive dye, aligning with the findings from prior research [1]. These results confirm that acid whey-derived BNC provides a suitable matrix for the development of intelligent food packaging labels with pH-responsive properties.

### 3.2. pH-Responsive Behavior of Anthocyanin and Label Integration in Packaging

Anthocyanin exhibits distinct color variations across different pH levels. As shown in Figure 1a, the anthocyanin solution appeared pink-red at pH 1 and 2, transitioned to purple at pH 3, and displayed shades of blue from pH 4 to 10. At pH 11, the solution turned light green. These color shifts were visually distinguishable, demonstrating the pH sensitivity of the dye.

To develop the pH-responsive labels, the acid whey-derived BNC material was processed through blending, bleaching, washing, dyeing, and drying. Figure 1b illustrates the integration of these labels into beef mince packaging, where both pink and purple labels were positioned at the center of the package on top of the mince samples. This placement allowed the real-time monitoring of meat freshness. Upon exposure to the beef mince, the labels gradually changed color, reflecting the variations in meat quality over time.

### 3.3. FTIR Spectroscopy Analysis of BNC, and Anthocyanin Incorporation

**Figure 2:**
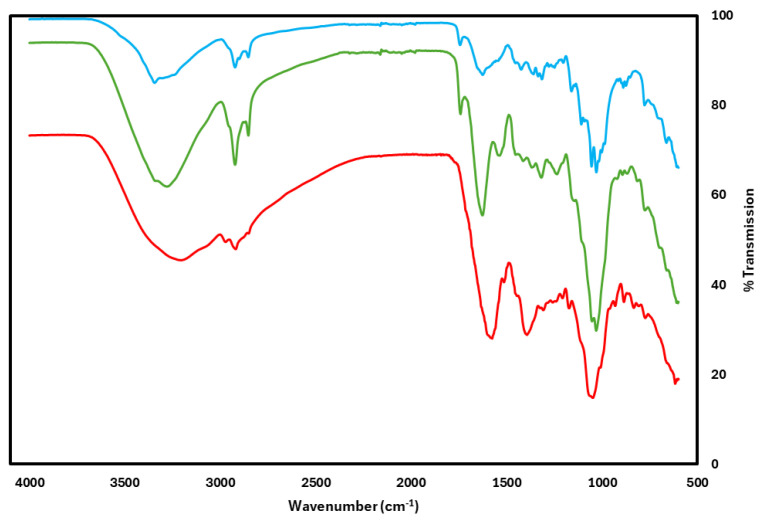
FTIR spectra of BNC labels. Blue line: BNC film; red line: anthocyanin extraction; green line: BNC label with anthocyanin extraction.

To verify the composition of BNC and the successful integration of anthocyanin into the pH-responsive labels, FTIR spectroscopy was conducted. Figure 2 presents the spectra of the BNC film, freeze-dried anthocyanin, and the anthocyanin-incorporating BNC label, covering a wavenumber range of 4000 to 600 cm^−1^. The analysis confirms the presence of cellulose, aligning with previous findings by Abol-Fotouh et al. [35] and Liu et al. [14]. As shown in Figure 2, the FTIR spectra exhibit characteristic cellulose peaks in the BNC material. The broad peak in the 3400–3200 cm^−1^ range corresponds to O-H stretching, while the peak around 2900 cm^−1^ is associated with C-H stretching [14]. Additionally, the peak near 1560 cm^−1^, observed in both the anthocyanin extract and the dyed BNC film, was probably related to aromatic ring stretching vibrations, and the slight increase in the intensity of the band around 1635 cm^−1^ was the confirmation of the successful incorporation of anthocyanin into the BNC matrix [2,36]. These results validate the structural integrity of the BNC film and its suitability as a carrier for pH-responsive dyes in intelligent food packaging applications.

### 3.4. TVBN, pH, and Microbial Growth as Indicators of Meat Spoilage

**Figure 3:**
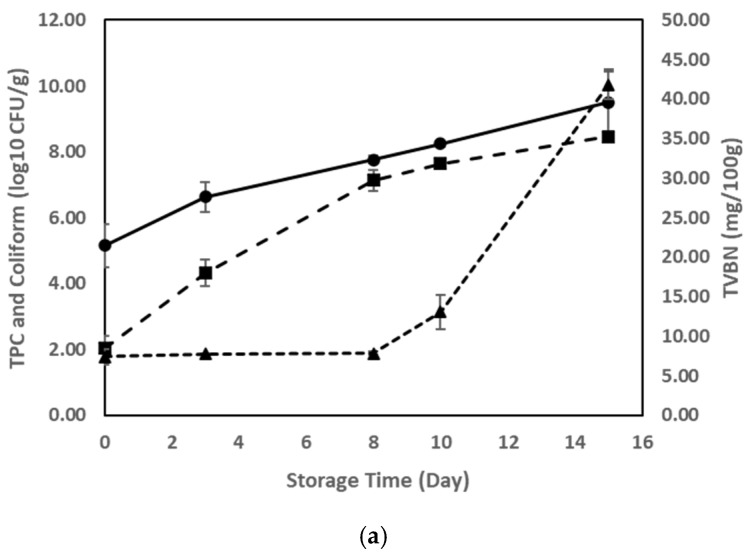

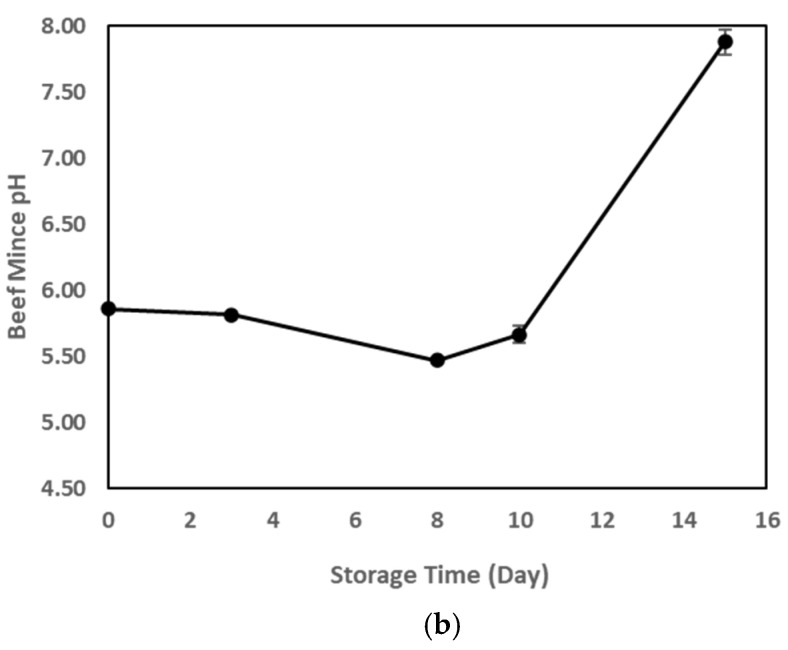
(**a**) Changes in TPC, coliform, and TVBN values of beef mince. Solid line (round mark): TPC; Long dash (square mark): coliform; Dash line (triangle mark): TVBN. (**b**) pH value of beef mince during storage.

TVBN levels are closely associated with the spoilage of meat products, as basic gases are generated due to biochemical and microbial activities. Figure 3a illustrates the increase in TVBN in correlation with the rise in Total Plate Count (TPC) and coliform levels. On day 0, coliform and TPC levels were approximately 10^2^ and 10^5^ CFU/g. By day 8, coliform levels increased to approximately 10^6^ CFU/g, while the TPC reached the upper 10^7^ CFU/g range. By day 15, coliform and TPC counts further rose to approximately 10^8^ and 10^9^ CFU/g, respectively. Over the 15-day storage period, TVBN values increased from 7.42 mg/100 g on day 0 to 7.84 mg/100 g on day 8 and further to 41.86 mg/100 g on day 15, marking a substantial deterioration in meat freshness. A noticeable acceleration in TVBN accumulation was observed after day 8, coinciding with increased microbial activity. Figure 3b shows the pH variations in the meat samples, which initially decreased from 5.86 on day 0 to 5.47 on day 8, followed by a rise from day 10, reaching 7.88 by day 15. These fluctuations align with microbial proliferation [37]. The concurrent changes in TVBN, pH, and microbial counts confirm the progressive decline in meat quality, reinforcing their reliability as indicators of spoilage [38].

### 3.5. TVBN Accumulation and Its Impact on pH-Responsive Label Color Changes

**Figure 4:**
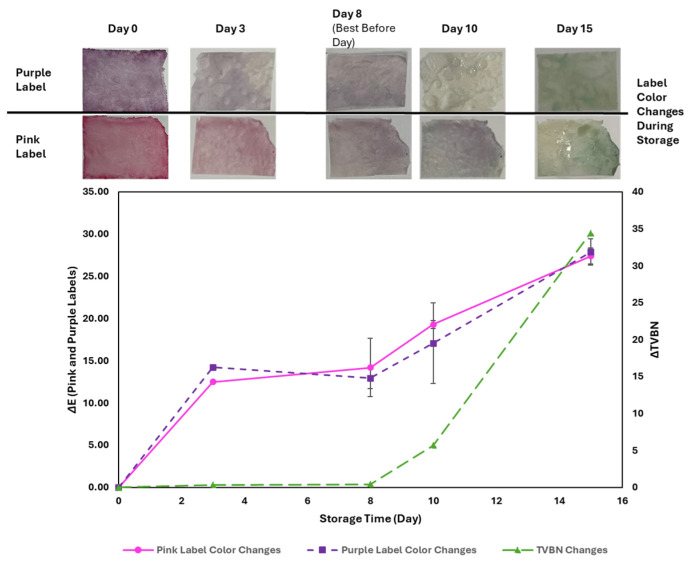
The color changes in the labels and ∆TVBN. Pink solid line (round mark): pink label color changes ∆E. Dash line (square mark): purple label color changes ∆E. Long dash line (triangle mark): ∆TVBN.

As TVBN accumulated in the package headspace, the pH-responsive labels underwent distinct color changes, reflecting the deterioration in freshness of the meat samples. The total color difference (∆E) increased progressively throughout the 15-day storage period. Figure 4 illustrates the sequential color shifts in both pink and purple labels. The pink label transitioned to light pink on day 3, turned purplish by day 8, became fully purple on day 10, and finally changed to light green on day 15. The purple label initially turned light purple on day 3 and darkened slightly on day 8 but exhibited less color variation than the pink label. By day 10, the purple label became lighter, before shifting to light green on day 15, mirroring the final color change of the pink label. The pink label provided more pronounced visual differences in the early stages of storage (day 0 to day 8), making it effective for detecting the initial freshness loss. Conversely, the purple label was more responsive between day 8 and day 10, making it useful for monitoring later-stage spoilage. Both labels turned light green after 15 days due to an excessive TVBN accumulation, indicating significant meat deterioration. The combination of pink and purple labels offers an enhanced ability to track quality changes near the best-before date, providing a more reliable freshness indicator.

### 3.6. Correlation Between ∆TVBN, TPC, Coliforms, and ∆E

As shown in Table 2, the total color difference (∆E) demonstrates a strong positive correlation with TVBN, coliform counts, and TPCs, confirming the effectiveness of the pH-responsive labels in monitoring meat spoilage. The TVBN analysis and microbial evaluations indicate a strong correlation between ∆E and ∆TVBN (r = 0.956 for pink, r = 0.993 for purple). Additionally, significant correlations were observed between ∆E and coliform counts (r = 0.933 for pink, r = 0.875 for purple), as well as between ∆E and TPC (r = 0.982 for pink, r = 0.950 for purple). These findings align with a previous study [2], reinforcing the potential of BNC-based pH-responsive labels as reliable indicators of meat freshness in intelligent food packaging systems.

## 4. Discussion

Acid whey, a by-product of cheese and yogurt production, is rich in nutrients, with its composition influenced by factors such as the origin of the milk, processing techniques, thermal treatments, and storage conditions [39]. Traditionally considered waste [40], its utilization as a fermentation substrate for bacterial nanocellulose production presents a sustainable and economically viable alternative. This approach not only mitigates dairy industry waste but also aligns with circular economy principles by repurposing an underutilized by-product into a high-value material [41,42]. The production of BNC from acid whey is an eco-friendly process, but optimizing fermentation parameters—including temperature, pH, reactor surface area, and duration—is crucial to improving yields and production efficiency [43]. Future research should explore the enhancement of acid whey-derived BNC production, possibly through the genetic modification of bacterial strains or process optimization techniques such as bioreactor design and nutrient supplementation [44].

The application of pH-responsive labels based on anthocyanin-incorporating BNC demonstrates a strong correlation with beef mince freshness, as evidenced by color shifts in response to the accumulation of TVBN. Anthocyanins, natural pigments derived from various plant sources [45], offer non-toxic and biodegradable alternatives for food packaging applications. *C. ternatea* anthocyanins, known for their stable color transformations [25], proved to be effective indicators of a deterioration in freshness in this study. The pink and purple labels responded differently to progression of spoilage, with pink being more visually distinct in the early stages and purple providing a clearer differentiation at the later stages. The combination of both labels enhances the ability to track freshness more effectively across the entire storage period, making them a promising tool for intelligent food packaging.

Given the success of this approach, future research could explore the incorporation of additional indicators to expand its applicability to other perishable food products, such as poultry, seafood, and dairy. Beyond pH-based indicators, integrating sensors for attributes like the oxidation state and microbial contamination could further refine real-time freshness monitoring [46,47,48]. Moreover, the scalability of this technology should be evaluated in commercial food packaging, including cost-effectiveness assessments, implementation processes, and consumer acceptance studies.

This research also supports broader sustainability goals by contributing to food waste reductions through cost-effective circular economy approaches. The real-time monitoring of food’s freshness can help consumers and retailers make informed decisions, thereby reducing premature disposal and extending products’ shelf life. Exploring the integration of BNC-based intelligent packaging within circular economy models could further enhance environmental benefits, creating a closed-loop system where by-products are continuously repurposed.

## 5. Conclusions

This study successfully demonstrates the development of pH-responsive labels using bacterial nanocellulose derived from acid whey and anthocyanin from *C. ternatea* for intelligent food packaging applications. The acid whey-based BNC film provides a sustainable and eco-friendly matrix for integrating natural pH-sensitive dyes, whilst repurposing a dairy industry by-product contributes to reducing waste. The color changes in the labels effectively reflect the freshness of beef mince over a 15-day storage period, with strong correlations observed between total color difference (∆E), TVBN, and microbial counts, confirming their reliability as freshness indicators. The pink label exhibited better sensitivity in the early spoilage stages, while the purple label was more effective in later stages. Combining both labels could improve the accuracy of freshness monitoring. These findings highlight the potential of acid whey-derived BNC films in intelligent food packaging. Future research should investigate label placement at multiple positions within the packaging to assess the robustness and general applicability of the system. In addition, extending this technology to a wider range of perishable foods, such as poultry, seafood, and dairy, will allow the assessment of its performance across diverse food matrices. The incorporation of indicators capable of detecting additional spoilage changes could further enhance the system. The further optimization of BNC’s production, mechanical properties, and scalability, along with consumer acceptance studies and life cycle assessments, will be key to advancing its commercial implementation. Collectively, this approach offers a promising pathway to reduce food waste, improve safety, and enable sustainable, real-time freshness monitoring in modern food supply chains.

## Figures and Tables

**Table 1:** Average yield and thickness of BNC film.

Parameter	Value (Mean ± SD)
Wet weight (g BNC/media L)	31.27 ± 9.60
Dry weight (g BNC/media L)	1.14 ± 0.39
Wet film thickness (mm)	2.75 ± 0.35

**Table 2:** Pearson correlation analysis of the color changes in the BNC labels and the beef mince freshness indicators.

Beef Mince Freshness Indicators	Pink Label ∆E	Purple Label ∆E
∆TVBN	0.956	0.993
Coliform Counts (Log10 CFU/g)	0.933	0.875
Total Plate Count (Log10 CFU/g)	0.982	0.950

## Data Availability

The original contributions presented in the study are included in the article, further inquiries can be directed to the corresponding author.

## Abbreviations

The following abbreviations are used in this manuscript:

BNC	Bacterial Nanocellulose
FTIR	Fourier-Transform Infrared
RO	Reverse Osmosis
SCOBY	Symbiotic Culture of Bacteria and Yeast
TAC	Total Anthocyanin Content
TPC	Total Plate Counts
TVBN	Total Volatile Basic Nitrogen
